# Association of Renal Dysfunction With Remote Diffusion-Weighted Imaging Lesions and Total Burden of Cerebral Small Vessel Disease in Patients With Primary Intracerebral Hemorrhage

**DOI:** 10.3389/fnagi.2018.00171

**Published:** 2018-06-07

**Authors:** Xu-hua Xu, Xiang-hua Ye, Jin-song Cai, Ting Gao, Guo-hua Zhao, Wen-ji Zhang, Lu-sha Tong, Feng Gao

**Affiliations:** ^1^Department of Neurology, The Fourth Affiliated Hospital, School of Medicine, Zhejiang University, Yiwu, China; ^2^School of Medicine, Zhejiang University, Hangzhou, China; ^3^Department of Neurology, The Second Affiliated Hospital, School of Medicine, Zhejiang University, Hangzhou, China; ^4^Department of Radiology, The Second Affiliated Hospital, School of Medicine, Zhejiang University, Hangzhou, China; ^5^Department of Radiology, The Fourth Affiliated Hospital, School of Medicine, Zhejiang University, Yiwu, China

**Keywords:** cerebral small vessel disease, remote DWI lesions, glomerular filtration rate, cystatin C, proteinuria, renal dysfunction

## Abstract

**Objective:** Remote diffusion-weighted imaging (DWI) lesions (R-DWIL) found in intracerebral hemorrhage (ICH) patients are considered as an additional marker of cerebral small vessel disease (cSVD). This study aimed to investigate the association of renal dysfunction and R-DWIL, as well as the total burden of cSVD on magnetic resonance imaging among patients with primary ICH.

**Methods:** One hundred and twenty-six consecutive patients were prospectively enrolled. R-DWIL on DWI, as well as other imaging markers of cSVD, including lacunes, white matter lesions, cerebral microbleeds, and enlarged perivascular spaces were rated using validated scales. Renal dysfunction was evaluated either by reduced estimated glomerular filtration rate (eGFR) or the presence of proteinuria or increased cystatin C.

**Results:** After adjustments for potential confounders by logistic regression, impaired eGFR [odds ratio (OR) 6.00, 95% confidence interval (CI) 1.73–20.78], proteinuria (OR 3.07, 95% CI 1.25–7.54) and increased cystatin C (OR 2.73, 95% CI 1.11–6.72) were correlated with presence of R-DWIL. A similar association was also found between cystatin C levels (OR 3.16, 95% CI 1.39–7.19), proteinuria (OR 2.79, 95% CI 1.34–5.83) and the comprehensive cSVD burden.

**Conclusions:** Renal dysfunction are associated with the presence of R-DWIL, and total burden of cSVD in patients with primary ICH.

## Introduction

Cerebral small vessel disease (cSVD) is an intrinsic disorder of the small perforating arterioles, and could increase the risks of stroke, mood disturbance, cognitive impairment, and balance disorder (Akoudad et al., 2015). Since it is difficult to directly visualize cSVD *in vivo*, the diagnosis of cSVD has mainly relied on the imaging methods of magnetic resonance imaging (MRI) findings including cerebral microbleeds (CMBs), white matter hyperintensities (WMH), enlarged perivascular spaces (EPVS), and lacunes (Pantoni, 2010).

Small vessel disease in the brain and kidneys may have a common pathogenesis source because these organs are closely related due to similar anatomical and vasoregulatory characters (Tasuku et al., 2009). Therefore, it is suggested that markers for early renal dysfunction may also serve as an early indicators of cerebral small vessel damage. Indeed, previous studies have demonstrated that renal dysfunction are associated with WMH, CMBs, EPVS, lacunes, and total cSVD load, a combined effect of all 4 markers (Ovbiagele et al., 2013; Oh et al., 2014; Staals et al., 2014; Zhang et al., 2014; Akoudad et al., 2015; Xiao et al., 2015; Banerjee et al., 2016; Yang et al., 2017). However, these studies mainly focused on patients of ischemic stroke, few on patients of primary ICH. Besides, remote diffusion-weighted imaging (DWI) lesions (R-DWIL) after ICH are sometimes considered another potential marker of cSVD (Werring et al., 2011; Kang et al., 2012; Wu et al., 2015). They are often subclinical but are associated with worsened clinical outcome (Garg et al., 2012; Kang et al., 2012). Currently, most studies defined renal dysfunction based on the conventional renal indicators, such as estimated glomerular filtration rate (eGFR) or proteinuria (Ovbiagele et al., 2013; Zhang et al., 2014; Xiao et al., 2015). Cystatin C has been proposed to be an alternative indicator for its higher sensitivity and stability (Ferguson et al., 2015). Thus, in this study, we aimed to investigate the association of renal dysfunction, using eGFR, proteinuria and cystatin C separately, with remote DWI lesions and total cSVD burden among the patients of primary ICH.

## Methods

### Study population

We prospectively recruited patients with primary ICH in the department of neurology at the second affiliated hospital of Zhejiang university from November 2016 to May 2017. Inclusion criteria was: ICH, MRI scan performed within 28 days of ICH onset. Exclusion criteria was: contraindications or refusal to MRI, secondary causes of ICH such as underlying aneurysm, vascular malformation, head trauma, venous infarction, hemorrhagic transformation of ischemic infarction, or tumor. Baseline demographic information, medical history, medications, and plasma C reactive protein (CRP) levels were collected at admission on all patients. Hematoma volume was calculated using the ABC/2 method based on initial computed tomography (CT) scan (Kothari et al., 1996). The study protocol was approved by the institutional Human Research Ethics Committee of the second affiliated hospital of Zhejiang university.

### Measurement of eGFR, proteinuria, and cystatin C

Estimated glomerular filtration rate (eGFR) was calculated individually by the following Chronic Kidney Disease Epidemiology Collaboration (CKD-EPI) equation for the Asian population: eGFR = 141 × min (serum creatinine/κ,1)α × max(serum creatinine/κ,1) −1.209 × 0.993Age × 1.018 [if female], where κ was 0.7 for females and 0.9 for males, α was −0.329 for females and −0.411 for males, min was the minimum of SCr/κ or 1, and max indicated the maximum of SCr/κ or 1 (Teo et al., 2011). A low eGFR level was defined as <60 mL/min/1.73 m^2^. Proteinuria was measured from the first morning urine sample on admission by dry chemistry method. Urine protein was recorded as negative (<15 mg/dL), trace (15–30 mg/dL), 1+ (30 mg/dL), 2+ (100 mg/dL), 3+ (300mg/dL), or 4+ (1,000 mg/dL). Serum cystatin C was measured from blood samples drawn upon admission with scattering nephelometry method.

### MRI protocol and assessment

MRI was performed on 1.5-Telsa (Sonata, Siemens, German) or 3.0-Telsa scanner (Signa HDxt, GE Healthcare, American) with standardized protocol consisted of axial T1-weighted, T2-weighted, T2 FLAIR, T2 star weighted angiography (SWAN), DWI, and apparent diffusion coefficient (ADC) sequences. Axial DWI sequences were acquired on 1.5T [repetition time (TR) 3,100 ms, echo time (TE) 84 ms, b = 0/1,000s/ mm^2^, 6-mm slice thickness, 0.5-mm gap, FOV 230 mm] or 3.0T scanner (TR 5,200 ms, TE 75 ms, b = 0/1,000 s/mm^2^, 6-mm slice thickness, 0.5-mm gap, FOV 240 mm) with different parameters. A neurologist of stroke specialty (L. S. T) and a radiologist (J. S. C) read the data of R-DWIL independently and reached consensus (Kappa = 1.0). The evaluations of EPVS, Lacunes, WMH and CMBs were performed by one experienced radiologist (W. J. Z) who was blind to the DWI study.

#### Assessment of EPVS, lacunes, WMH, and CMBs

EPVS were defined as ≤2 mm round or linear CSF isointense lesions (T2-hyperintense and T1/FLAIR hypointense with respect to brain) along the course of penetrating arteries. They were distinguished from lacunes by the size difference (lacunes > 2 and ≤15 mm) and the surrounding hyperintensity rim in FLAIR (Maclullich et al., 2004; Hansen et al., 2015). EPVS were counted on the brain slice showing the greatest extent of EPVS in the basal ganglia (BG) and centrum semiovale (CSO) regions separately (Charidimou et al., 2014). WMH was rated visually on axial FLAIR images on the 4-point Fazekas scale, modified to include both periventricular white matter (PVWM) and deep white matter (DWM) (Gao et al., 2015). CMBs were defined as rounded or circular foci with low signal intensity on SWAN-sequences with a maximum diameter of 10 mm. Mimics of CMBs such as calcifications, cavernomas, small pial blood vessels, partial volume artifacts were always considered, and, whenever identified, excluded from analysis (Greenberg et al., 2009).

#### Assessment of R-DWIL

R-DWIL were defined as hyperintense lesions on DWI sequence, with corresponding dark areas on ADC maps. R-DWIL were distant from initial hematoma, and DWI lesions close to an hematoma (<20 mm) were excluded (Wu et al., 2015).

#### Assessment of total SVD burden

Based on the method described in recent literatures (Staals et al., 2014, 2015; Wiseman et al., 2016), we rated the total MRI burden of cSVD on an ordinal scale from 0 to 4, by counting the presence of each of the 4 MRI features of SVD. A point was awarded for each of the following: presence of lacunes and CMBs were defined 1 point, separately. Presence of EPVS was counted if there were moderate to extensive (10–25 or >25) EPVS in the basal ganglia (1 point if present). Presence of WMH was defined as either confluent deep WMH (Fazekas score 2 or 3) or irregular periventricular WMH extending into the DWM (Fazekas score 3) (1 point if present).

### Statistical analysis

For purposes of these analyses, proteinuria grades were dichotomized into a binary variable by incorporating no proteinuria and trace into a single category and proteinuria grades 1+, 2+, 3+, and 4+ into another category. Cystatin C was divided into two groups based on the 75% quartile values (<1.15 mg/L *vs* ≥1.15 mg/L). eGFR was divided into two groups based on <60 mL/min/1.73 m^2^ vs. ≥60 mL/min/1.73 m^2^. Differences in dichotomous variables were analyzed using χ2 analysis or the Fisher exact test. Student *t* test or the Wilcoxon rank-sum test was used to analyze differences in the mean or median of continuous variables between groups. Colinearity between the variables were assessed by the Pearson correlation analysis (cystatin C and eGFR, cystatin C and proteinuria). To examine the association between three renal markers and R-DWIL or total burden of cSVD, we divided patients into 2 groups based on the presence vs. absence of R-DWIL, or 5 groups based on the scores of cSVD, then conducted multivariable binary or ordinal logistic regression. All multivariable analyses were first adjusted for age and sex (Model 1) and additionally adjusted for variables including age, sex, hypertension, diabetes mellitus, previous ICH, previous stroke/transient ischemic attack (stroke/TIA), current smoker, alcohol, antiplatelets, antihypertensive drugs and CRP (Model 2). A *p*-value < 0.05 was considered to be statistically significant. All statistical analyses were carried out using SPSS (IBM SPSS Statistics 24.0).

## Results

### Baseline characteristics

**Figure 1:**
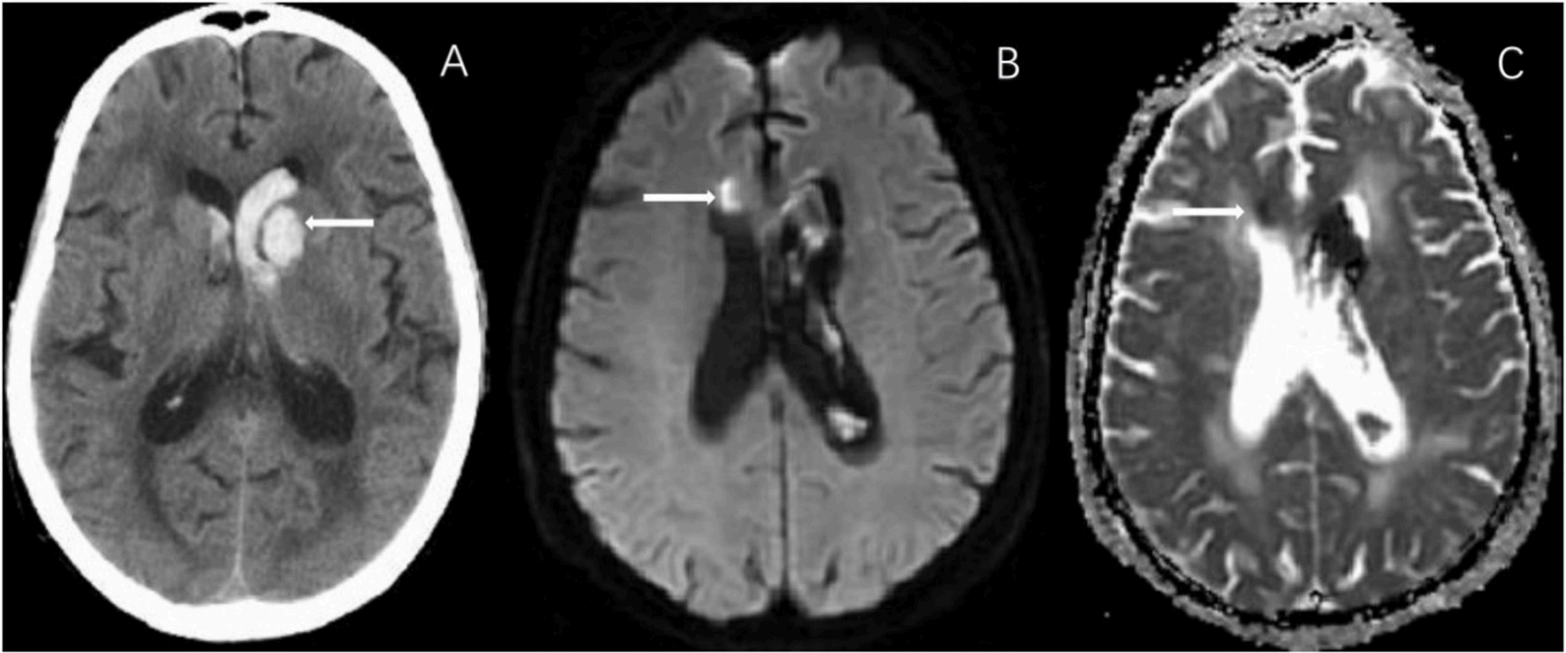
In a 75-year-old female with left basal ganglia hemorrhage **(A)**, diffusion weighted imaging (DWI) shows a small remote ischemic lesion on right frontal angle **(B)**, with corresponding low signal intensity in apparent diffusion coefficient (ADC) map **(C)**.

**Figure 2:**
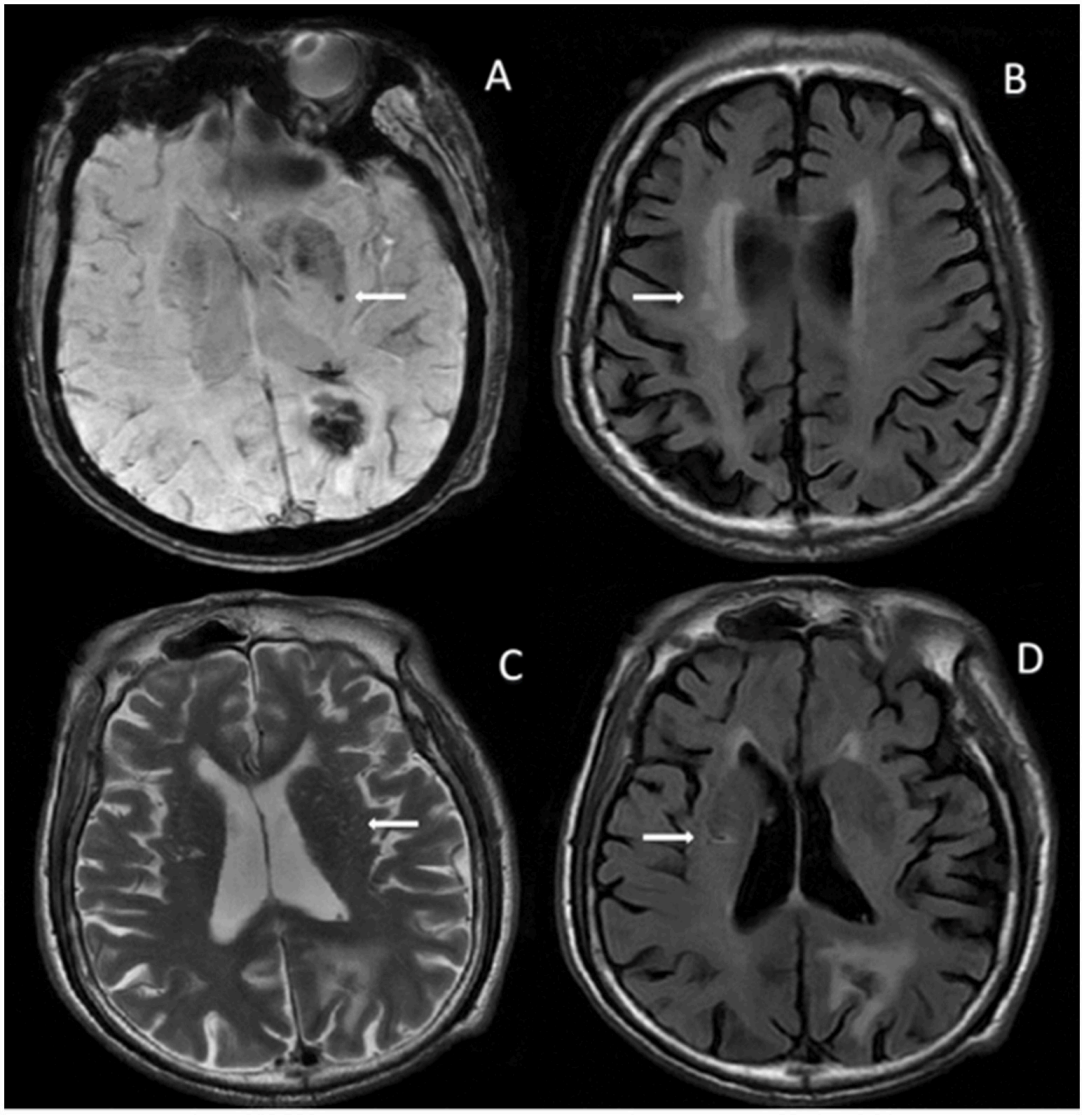
In an 80-year-old man with left occipital hematoma, MRI shows all 4 markers involved in total cSVD burden. **(A)** SWAN shows CMBs in left basal ganglia. **(B)** FLAIR image shows PWMH in bilateral side. **(C)** T2-sequence shows EPVS in bilateral basal ganglia. **(D)** FLAIR image shows lacune in right para-lateral ventricle. No R-DWIL was found on MRI (not shown). eGFR in this patient was 85.2 mL/min/1.73 m^2^, proteinuria was 2+, and cystatin C was 2.07 mg/L. CMBs, cerebral microbleeds; EPVS, enlarged perivascular spaces; FLAIR, fluid-attenuated inversion recovery; MRI, magnetic resonance imaging; SWAN, star weighted angiography; PWMH, periventricular white matter hyperintensity; R-DWIL, remote DWI lesions; eGFR, estimated glomerular filtration rate.

Finally, 126 patients (mean age, 61.8 ± 13.2 years; 56.3%male) with primary ICH were enrolled in this study. The median time from ICH onset to admission was 1.5 days [interquartile range (IQR), 1–3 days], and from ICH onset to MRI scan was 6 days (IQR, 4–8 days). Median baseline hematoma volume was 8.6 cm^3^ (IQR, 3.2–18.3 cm^3^). Median plasma CRP was 5 mg/L (IQR, 2–10mg/L). Table 1 presents the clinical characteristics of all included patients according to categories of three renal indicators, eGFR, proteinuria, and cystatin C. In this study, 35 (27.8%) patients had cystatin C levels≥1.15 mg/L, 38 (30.2%) patients had proteinuria (1+, 2+, 3+, 4+), 12 (9.5%) patients had both lower eGFR levels (eGFR < 60 mL/min/m^2^) and higher cystatin C levels, 19 (15.1%) patients had both higher cystatin C levels and proteinuria, 8 (6.3%) patients had abnormality of 3 renal indicators. Among the total 126 patients, 29 patients (23.0%) had remote ischemic lesions (Figure 1). For cSVD burden, 21 (16.7%) patients had a total cSVD score = 0, 25 (19.8%) patients had a total cSVD score = 1, 32 (25.4%) patients had a total cSVD score = 2, 28 (22.2%) patients had a total cSVD score = 3, and 20 (15.9%) patients had a total cSVD score = 4 (Figure 2).

**Table 1:** Characteristics of Patients by eGFR, Proteinuria, and Cystatin C.

	**eGFR ≤ 60 mL/min/1.73 m**^**2**^			**Proteinuria (1+, 2+,3+,4+)**			**Cystatin C≥1.15 mg/L**		
	**Yes**	**No**	***P*-value**	**Yes**	**No**	***P*-value**	**Yes**	**No**	***P*-value**
N, %	12(9.5)	114(90.5)		38(30.2)	88(69.8)		35(27.8)	91(72.2)	
Age years, mean(SD)	65.8(16.3)	61.4(12.9)	0.266	63.3(14.0)	61.1(12.9)	0.405	66.6(14.9)	59.9(12.1)	0.011
Male, %	7(58.3)	64(56.1)	0.884	19(50.0)	52(59.1)	0.345	24(68.6)	47(51.6)	0.086
Hypertension, %	10(83.3)	81(71.1)	0.508	30(78.9)	61(69.3)	0.268	61(67.0)	30(85.7)	0.036
Diabetes mellitus, %	3(25.0)	9(7.9)	0.089	4(10.5)	8(9.1)	0.345	7(7.7)	5(14.3)	0.312
Previous ICH, %	2(16.7)	8(7.0)	0.243	2(5.3)	8(9.1)	0.722	3(8.6)	7(7.7)	1.000
Previous stroke/TIA, %	2(16.7)	9(7.9)	0.281	4(10.5)	7(8.0)	0.733	7(20.0)	4(4.4)	0.010
Antiplatelets, %	1(8.3)	4(3.5)	0.399	1(2.6)	4(4.5)	1.000	2(5.7)	3(3.3)	0.617
Antihypertensive drugs, %	6(50.0)	49(43.0)	0.641	18(47.4)	37(42.0)	0.580	23(65.7)	32(35.2)	0.002
Smoker, %	6(50.0)	26(22.8)	0.074	12(31.6)	20(22.7)	0.295	13(37.1)	19(20.9)	0.060
Alcohol, %	0(0.0)	21(18.4)	0.215	5(13.2)	16(18.2)	0.487	5(14.3)	16(18.2)	0.487
CRP mg/L, median(IQR)	2.8(1.7–41.2)	5.0(2.0–10.0)	0.380	3.5(1.6–10.1)	5.4(2.7–10.2)	0.818	5.0(1.9–7.5)	4.9(2.0–10.7)	0.573
Hematoma Volume cm3, median(IQR)	5.1(3.0–13.0)	9.1(3.6–18.6)	0.905	7.4(3.0–16.9)	9.0(4.2–19.3)	0.283	5.3(2.7–11.7)	10.3(4.2–19.3)	0.283
Lobar Hematoma, %	2(16.7)	31(28.2)	0.510	10(26.3)	23(27.4)	0.902	10(26.3)	23(27.4)	0.902
Deep Hematoma, %	10(83.3)	80(72.7)	0.730	28(73.7)	62(73.8)	0.988	28(73.7)	62(73.8)	0.988
Lacune, %	8(66.7)	58(51.3)	0.312	26(70.3)	40(45.5	0.011	25(73.5)	41(45.1)	0.005
WMH, %	3(25.0)	39(34.5)	0.749	17(45.9)	25(28.4)	0.058	17(50.0)	25(27.5)	0.018
CMBs, %	22(78.6)	63(65.6)	0.194	28(75.7)	57(65.5)	0.265	27(79.4)	58(64.4)	0.109
EPVS, %	9(75.0)	76(67.9)	0.751	20(54.1)	36(40.9)	0.177	23(67.6)	33(36.3)	0.002
R-DWIL, %	7(58.3)	22(19.3)	0.006	14(36.8)	15(17.0)	0.015	14(36.8)	15(17.0)	0.015
cSVD Score, %			0.316			0.006			0.000
0	1(8.3)	20(17.5)		2(5.3)	19(21.6)		2(5.7)	19(20.9)	
1	1(8.3)	24(21.1)		7(18.4)	18(47.4)		4(11.4)	21(23.1)	
2	3(25.0)	29(25.4)		8(21.1)	24(63.2)		5(14.3)	27(29.7)	
3	7(58.3)	21(18.4)		12(31.6)	16(42.1)		14(40.0)	14(15.4)	
4	0(0.0)	20(17.5)		9(23.7)	11(28.9)		10(28.6)	10(11.0)	

*SD, indicates standard deviation; eGFR, estimated glomerular filtration rate; N, number; ICH, intracerebral hemorrhage; TIA, transient ischemic attack; CRP, C-reactive protein; IQR, interquartile range; CMBs, cerebral microbleeds; WMH, white matter hyperintensity; EPVS, enlarged perivascular spaces; R-DWIL, remote DWI lesions; cSVD, cerebral small vessel disease*.

### Association of eGFR and proteinuria with R-DWIL and total burden of cSVD

Baseline demographic and clinical characteristics between those with eGFR < 60 mL/min/m^2^ vs. those ≥ 60 mL/min/m^2^ are shown in Table 1. Most of these characteristics were comparable, but patients with lower eGFR levels were significantly more likely to have the presence of R-DWIL, but this correlation was not found with cSVD scores. The presence of proteinuria was found to be related to R-DWIL, as well as to the presence of lacunes and increased cSVD burden.

### Association of cystatin C with eGFR and proteinuria

**Figure 3:**
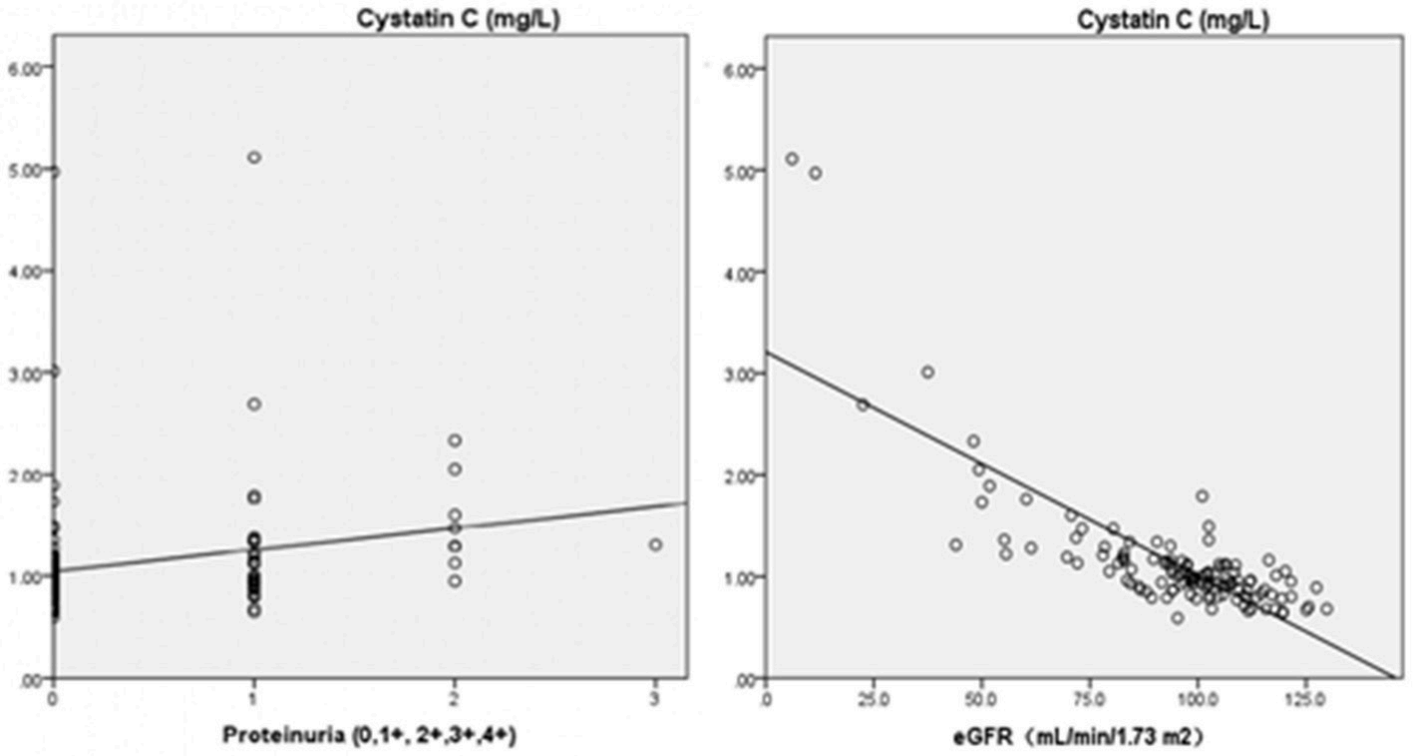
Pearson's correlation analysis between cystatin C and eGFR or proteinuria (Cystatin C and eGFR, *r* = −0.80, *p* = 0.000; Cystatin C and proteinuria, *r* = 0.218, *p* = 0.015). ° Indicates data from patients; eGFR, estimated glomerular filtration rate.

As two continuous variables, the association between cystatin C and eGFR in the patients with primary hemorrhage showed a strong linear correlation (*r* = −0.80; *p* = 0.000). We also found a similar association between cystatin C and proteinuria grades (*r* = 0.218; *p* = 0.015; Figure 3).

### Association of cystatin C with R-DWIL and total burden of cSVD

Table 1 presents the characteristics of the study population stratified by the 75% quartile values of cystatin C. Higher cystatin C levels showed significant associations with some basal information like increasing age, a history of hypertension, previous stroke/TIA, previous usage of antihypertensive drugs. As for the presence of R-DWIL and total cSVD burden, cystatin C continued to show strong correlations. Besides, cystatin C was also related with other cSVD markers like the presence of lacunes, WMH and EPVS.

### Multivariable analysis of association of eGFR, proteinuria and cystatin C with R-DWIL and total burden of cSVD

Table 2 summarizes the results of the binary or logistic regression of the R-DWIL or the total burden of cSVD. Low eGFR, high cystatin C and elevated protein in the urine were significantly associated with R-DWIL, with adjustment for age and sex (model1). After adjusting for potential confounders (age, sex, hypertension, diabetes mellitus, previous ICH, previous stroke/TIA, smoker, alcohol, antiplatelets, antihypertensive drugs, and CRP) (model 2), low eGFR [odds ratio (OR)6.00, 95% confidence interval (CI) 1.73–20.78], high cystatin C (OR 2.73, 95% CI 1.11–6.72) and proteinuria (OR 3.07, 95% CI 1.25–7.54) were still related with presence of R-DWIL (Table 2). For the total cSVD burden, high cystatin C, and elevated proteinuria were significantly associated with the severity of cSVD in model 1. And these two measures of renal dysfunction remained as independent predictors after adjusting for all confounding variables (OR 3.16, 95% CI 1.39–7.19 for cystatin C and OR 2.79, 95% CI 1.34–5.83 for proteinuria).

**Table 2:** Association of eGFR, Proteinuria and Cystatin C Levels with R-DWIL and Total Burden of cSVD.

	**R-DWIL OR (95%CI)**		**cSVD Burden OR (95%CI)**	
	**Model 1**	**Model 2**	**Model 1**	**Model 2**
eGFR (≤ 60 mL/min/1.73 m^2^ vs >60 mL/min/1.73 m^2^)	5.86(1.70–20.20)^**^	6.00(1.73–20.78)^**^	—	—
Cystatin C(≥1.15 mg/L vs < 1.15 mg/L)	2.77(1.16–6.63)^*^	2.73(1.11–6.72)^*^	3.61(1.69–7.72)^**^	3.16(1.39–7.19)^**^
Proteinuria (1+,2+,3+,4+ vs 0,+/−)	2.84(1.20–6.72)^*^	3.07(1.25–7.54)^*^	2.69(1.33–5.44)^**^	2.79(1.34–5.83)^**^

*Model 1: bivariate logistic regression analyses with adjustment for age and sex. Model 2: bivariate logistic regression analyses with adjustment for age, sex, hypertension, diabetes mellitus, previous ICH, previous stroke/TIA, smoker, alcohol, antiplatelets, antihypertensive drugs, and CRP. CI indicates confidence interval; OR, odds ratio; eGFR, estimated glomerular filtration rate; cSVD, cerebral small vessel disease; RIL, remote ischemic lesions*;

* *, p < 0.05*;

** *, p < 0.01; —, p ≥ 0.05*.

## Discussion

In this study, we demonstrated that decreased eGFR, as well as elevated urine protein and high cystatin C level were associated with the presence of R-DWIL in primary ICH patients, and high cystatin C level and elevated urine protein were also associated with increased cSVD burden in these patients, demonstrated by the cooccurrence of four different MRI markers.

The association between renal dysfunction with the presence and extent of WMH or CMBs or EPVS or lacunes has been investigated, as well as with total cSVD burden among patients with ischemic stroke (Oh et al., 2014; Staals et al., 2014; Zhang et al., 2014; Akoudad et al., 2015; Xiao et al., 2015; Banerjee et al., 2016; Yang et al., 2017). However, no studies are available to date investigating the association of renal dysfunction with total cSVD burden in primary ICH patients, in which cSVD accounts for a great portion of etiology. Our study demonstrated similar association between proteinuria and cSVD. However, in contrast to previous studies in ischemic stroke, we did not find an association between eGFR and cSVD. Potential explanations for the observation are first, a lack of statistical power in our study due to low incidence of renal dysfunction based on eGFR. Then differences in the setting of study populations yield different subtypes of cSVD, thus resulting in different imaging markers of cSVD. It is well known that primary ICH is mainly related with two types of cSVD, cerebral amyloid angiopathy (CAA) and hypertensive angiopathy (HA), while subtypes of cSVD in ischemic stroke relatively have greater heterogeneity. Besides, pathophysiologic change during the acute phase of stroke also differ between two subtypes of stroke, which may affect renal markers differently. It has been reported that occurrence of acute renal failure is more common in patients with ICH compared with those with other stroke subtypes (Saeed et al., 2015).

Remote DWI lesions (R-DWIL) have been reported in 11.1–41% of patients with primary ICH (Xu et al., 2017). According to our observation, 23% patients with acute primary ICH had R-DWIL, corroborating previous studies. These R-DWIL sometimes have been considered an additional marker of cSVD (Werring et al., 2011; Kang et al., 2012; Tsai et al., 2014; Wu et al., 2015). However, R-DWIL could have other mechanisms beyond cSVD, such as cerebral atherosclerosis, blood pressure lowering, and remote extension of hematoma (Xu et al., 2017), which may render it difficult to reliably use them as a specific biomarker of cSVD. On the basis of this consideration, we did not include R-DWIL in the total cSVD score. As far as we are aware, few study has examined the link between renal dysfunction and R-DWIL among primary ICH patients. Similar to the association between renal dysfunction and cSVD burden, we also found a strong association of eGFR and proteinuria with the presence of R-DWIL, further highlighting the close relationship of cSVD with kidney disorders. Besides, as R-DWIL can be especially predictive of functional disability at 3 months and future ischemic stroke and recurrent ICH risk (Gioia et al., 2015), observing a relationship between renal dysfunction and R-DWIL among primary ICH patients could be an affirmation of renal dysfunction as a prognosticator in ICH patients at risk for poor outcome and developing ischemic stroke or recurrent ICH (Molshatzki et al., 2011).

Cystatin C is a cysteine proteinase inhibitor that is released at a constant rate from all nucleated cells. It is filtrated freely by the glomerulus and is almost completely reabsorbed and catabolized in the renal tubules (Newman, 2002). As less affected by extrarenal factors compared to creatinine and proteinuria, cystatin C has been proposed to be a sensitive and reliable marker of kidney function (Ferguson et al., 2015). The notion is also supported by our observation that patients who had eGFR levels < 60 mL/min/m2, all had higher cystatin C levels. Currently, there are only a few studies on the association between cystatin C and markers of cSVD (Wada et al., 2010; Oh et al., 2014; Yang et al., 2017). Our results showed that cystatin C was the most powerful indicator for total cSVD burden among the three renal markers, consistent with previous studies. Beyond the role as a reliable marker for kidney function, cystatin C has also received much attention due to its multiple biological activities involved in neurological diseases. In ischemic WMH, the secretion of cystatin C is increased in the regressive astrocytes in response to the stimuli of proteases and inflammatory cytokines, possibly a self-defense to white matter degeneration (Umegae et al., 2008). Cystatin C also accumulates in the smooth muscles of the cerebral penetrating arterioles in patients with CAA and Alzheimer's dementia, which might facilitate the dysregulation of the composition of the basement membrane, and the disruption of smooth muscle layer (Levy et al., 2001, 2006). Moreover, cystatin C regulates the extracellular matrix turnover, and an imbalance between cysteine cathepsins and cystatin C could promote the progression of cerebral aneurysms (Aoki et al., 2008). On consideration of these factors, it seems reasonable to consider cystatin C a better indicator for cSVD than other renal markers. However, cystatin C failed to show a most significant association with the presence of R-DWIL, while eGFR did. This may be partly a consequence of the way to categorize cystatin C. As there is no standard threshold to define renal dysfunction based on cystatin C, renal dysfunction was just set based on the 75% quartile values of cystatin C without further confirmation.

Inflammation is a major feature of primary ICH pathology (Di Napoli et al., 2014). The hematoma could induce not only local inflammatory response contributing to secondary brain injury after ICH, but also systemic state of inflammation through mechanisms including immunological, endothelial dysfunction, and coagulopathy, leading to multiple organ failure (Malham and Souter, 2001). It is possible that the inflammation might affect both kidney function and cerebral circulation. Among Inflammation markers, plasma CRP has been reported particularly associated with early hematoma growth and early neurological worsening, as well as poor outcomes after ICH (Di Napoli et al., 2012, 2014). Thus, we also assessed the inflammatory status of the patients using plasma CRP. The CRP levels were lower than those in previous studies (Di Napoli et al., 2012, 2014). This may be due to less severe patients and more delay time to blood sampling in our study. Besides, we could not be able to exclude cases with confounding factors like accompanying infections. As the relationship between renal markers and total burden of cSVD remained same after adjustment for CRP, it is unlikely that underlying inflammation is the only explanations for the association between renal dysfunction and cSVD in the present observation.

Although not fully elucidated, the link between renal dysfunction and cSVD is assumed to be related with anatomical and functional similarity between glomerular afferent arterioles of juxtamedullary nephrons and cerebral perforating arteries. In the systemic circulation, the brain and kidneys are unique for their high-volume blood flow throughout systole and diastole, whereas their small arteries are exposed to high pulsatile pressure on account of vasodilation upstream (O'Rourke and Safar, 2005). Therefore, small vessels in these two organs are more susceptible to insults that may augment fluctuations of pressure and flow, compared with small vessels in other organs, which are protected by relatively intense vasoconstriction upstream (O'Rourke and Safar, 2005). In fact, kidney disease and cSVD are usually found to have common pathologic basis-lipohyalinosis (Roger and Duvoisin, 1969). Besides, renal dysfunction might also contribute to cSVD via inducing nitric oxide deficiency. Nitric oxide plays an important role in the proliferation of smooth muscle and maintaining microcirculation and blood brain barrier function. The superoxide scavenging of nitric oxide increases pro-inflammatory and pro-thrombotic properties, and eventually leads to generalized endothelial dysfunction and systemic vascular remodeling (Faraci and Brian, 1994). In addition, activation of the reninangiotensin system, as well as sodium retention and increased catecholamine levels, found in patients with renal dysfunction, could also lead to elevation of blood pressure, resulting in a loss of blood flow auto-regulation (Masuo et al., 2010).

This study has several limitations. First, our cohort may favor milder patients, as patients with more severe hemorrhages may have undergone neurosurgery or unstable for MRI. Second, although it is widely accepted that studies on cSVD are needed to take into account the total cSVD burden on imaging, there are no universal approaches to assess it. The approach we applied with was first attempted by Staals et al. (2014), then widely used in the studies focusing on the relationship between cSVD and systemic lupus erythematosus, cognitive impairment and aortic atheroma (Staals et al., 2015; Song et al., 2016; Wiseman et al., 2016). However, whether this approach could capture total brain damage resulting from cSVD still need further validation. Third, it is noteworthy that three renal markers were just measured once on admission, these markers could still be evolving. Besides, proteinuria could also be affected by urinary tract infections easily. Therefore, it is better to measure these markers more times or to collect urinary protein in 24 h to avoid potential bias. It also should be pointed out the inability to attribute renal dysfunction to pre-existing renal failure or acute kidney injury, as two cases may have different ways to influence brain vessels. Finally, we did not classify the etiology of ICH due to sample size. As mentioned before, primary ICH is closely related with two subtypes of cSVD, that is CAA and HA. Among them, HA is especially associated with kidney disease for their common vascular pathological basis-hyalinosis (Hassan et al., 2004). The deficiency of ICH classification may have limited our ability to examine the association between renal dysfunction and cSVD.

Since cSVD is a common cause of cognitive impairment, balance disorder and stroke syndromes, future studies should include these targets to assess long-term effects of renal dysfunction. Besides, dynamic observation is also needed to further clarify the relationship between renal dysfunction and cSVD burden on MRI. At last, considering eGFR, cystatin C and proteinuria might reflect a more generalized process indicative of underlying vascular damage, future studies may focus on whether drugs controlling renal dysfunction like RAS modulators have therapeutic implication for treatment of cSVD and whether these renal markers could serve as efficacy markers for treatment of cSVD.

In conclusion, our data suggest that renal dysfunction are associated with the presence of R-DWIL and accumulated burden of cSVD in patients with primary ICH. Our findings could provide a new idea for clinic forecasting of R-DWIL and cSVD burden in ICH patients.

## Author contributions

XX composed the manuscript. XY collected clinical records. JC assessed the radiographic results. TG helped to collect clinical records. GZ helped to analyze data statistically. WZ helped to assess radiographic results. LT and FG supervised the whole study.

### Conflict of interest statement

The authors declare that the research was conducted in the absence of any commercial or financial relationships that could be construed as a potential conflict of interest.
